# Diabetes Has Minimal Effect on High Gastrointestinal Symptom Burden in Exocrine Pancreatic Insufficiency Based on EPI/PEI-SS Scores

**DOI:** 10.3390/jcm14155422

**Published:** 2025-08-01

**Authors:** Dana M. Lewis

**Affiliations:** 1 OpenAPS, Seattle, WA 98101, USA; Dana@OpenAPS.org; 2 Department of Medicine, University of Otago, Christchurch 8011, New Zealand

**Keywords:** diabetes, exocrine pancreatic insufficiency, EPI, PEI, gastrointestinal, PERT, enzyme, pancreatic exocrine insufficiency, T1D, T2D

## Abstract

**Background:** Exocrine pancreatic insufficiency (EPI or PEI) may be prevalent in as many as 3 of 10 people with diabetes due to exocrine pancreatic function being reduced as early as the time of diagnosis. EPI can be treated with pancreatic enzyme replacement therapy (PERT), but the symptom burden of EPI remains high and improved screening and diagnosis methods are needed. **Methods:** An online survey (n = 324) evaluated the gastrointestinal symptom experiences of people with (n = 155) and without (n = 169) EPI using a novel symptom tool, the Exocrine Pancreatic Insufficiency Symptom Score (EPI/PEI-SS). A large sub-group (n = 120) of people with diabetes with EPI (Type 1, n = 14, Type 2, n = 20) or without EPI (Type 1, n = 78; Type 2; n = 6) was characterized and compared to those without diabetes (n = 204) in a sub-analysis of the larger EPI/PEI-SS study. **Results:** The symptom burden of EPI is similar, irrespective of diabetes. Like those without diabetes, people with type 1 diabetes with EPI had a statistically significant (*p* < 0.001) higher mean score (range 0–225) on the EPI/PEI-SS (100.86, SD: 48.92) than people with T1D without EPI (31.59, SD: 28.25), distinct from other GI conditions (*p* < 0.001). Similar patterns occurred in those with T2D. **Conclusions**: High EPI/PEI-SS scores seem to distinguish between likely EPI and other GI conditions among people with diabetes, and the EPI/PEI-SS should be further studied as a possible screening method for EPI at a population level. It should also be evaluated as a tool to aid individuals with diabetes in tracking changes to EPI symptoms over time based on PERT titration.

## 1. Introduction

Type 1 diabetes is perceived to be a disease of the endocrine pancreas, but research increasingly shows that pancreatic size [1] and exocrine pancreatic function are reduced in people with Type 1 diabetes at diagnosis [2]. Reduced exocrine function can result in exocrine pancreatic insufficiency (EPI or PEI) [3], which occurs when the pancreas no longer produces sufficient enzymes to successfully digest food on its own [4]. EPI is often misdiagnosed [5]. EPI is treated with pancreatic enzyme replacement therapy (PERT) [6], which is an oral enzyme supplementation based on food intake [7]. However, diagnosis of EPI can be challenging [8] due to perceptions related to the sensitivity and specificity of fecal elastase tests [9]. As a result, clinicians may dismiss results that indicate EPI [10], deviating from best practice clinical guidelines [6] that indicate individuals with lowered elastase should be prescribed a trial of PERT.

Compounding these issues is a lack of awareness about the prevalence of EPI, particularly among people living with diabetes [11]. Population estimates suggest it is mathematically improbable [12] that chronic pancreatitis or cystic fibrosis could be the likely biggest drivers of EPI [13]. Instead, because of the prevalence of diabetes (all types), people with diabetes likely make up the largest population of people living with EPI, and a recent systematic review [14] shows a possible prevalence of around 29% (T1D) and 33% (T2D) [14] (likely higher than celiac and gastroparesis combined). While data doesn’t necessarily support asymptomatic screening for EPI, people with diabetes who present with gastrointestinal symptoms should be considered for screening for EPI [2].

Recently, a symptom score was developed with the goal of identifying symptoms characteristic of EPI and assessing this score’s ability to differentiate those from everyday gastrointestinal (GI) symptoms and symptoms that typically overlap with other GI-related conditions [15,16]. The Exocrine Pancreatic Insufficiency Symptom Score (EPI/PEI-SS) includes fifteen individual symptoms that are scored based on the frequency and severity of each symptom. The EPI/PEI-SS was tested with an initial pilot (n = 15) followed by a larger survey (n = 324), including both the general population (n = 169) and in people with EPI (n = 155) [17].

A large population of people with T1D (n = 92) contributed to the survey, which provides an opportunity to assess the presence and distinction of GI symptoms more deeply with (n = 14) and without EPI (n = 78), and subsequently with and without other GI-related conditions such as gastroparesis and celiac, which are more widely studied in diabetes. While the overall results of the study of the EPI/PEI-SS are covered in a separate paper [17], this paper analyzes the differences among people with diabetes (both Type 1 and Type 2 diabetes) with and without EPI and with and without other GI-related conditions to articulate the common GI symptom burden that many people with diabetes are experiencing. These results were presented at ADA’s 2024 Scientific Sessions [18].

## 2. Materials and Methods

### 2.1. Survey Development and Recruitment

The development of the Exocrine Pancreatic Insufficiency Symptom Score (EPI/PEI-SS) is covered more deeply in a previous paper [17]. The score was developed by a person living with EPI based on existing literature [19], patient-reported experiences, and feedback from an initial pilot (n = 15) including that of other individuals (n = 7) living with EPI. Fifteen symptoms were grouped into three categories (abdominal-, toilet- and food-related symptoms) and the score was generated by multiplying the frequency (0–5 possible) against the severity (0–3 possible), for a total individual symptom score of max 15 and an overall total score ranging from 0 to 225 possible. It addresses gaps in previous symptom scores [20,21] which have only been validated among people with EPI and CF or CP [22] or pancreatectomy [21] and have so far not been demonstrated to be effective or validated among sub-populations of EPI such as people with diabetes [23] or who had bariatric surgery [24].

This was a community-designed and -led research study, and best practices of ethical research were followed mirroring previous studies within and for this community [25], including not collecting any identifying information and preserving the inability to contact participants due to anonymity. Participants were aware of who designed the survey (DL) and were informed that they had no obligations to fill out the survey and could stop at any time. Participants consented to the survey after reviewing text that indicated the study purpose, voluntary nature, data handling, and the absence of any identifiers being collected. No direct identifiers or indirect identifiers were collected. This anonymous, minimal-risk survey research met the criteria for exemption under the U.S. Common Rule (§46.104 d (2)) [26] and HIPAA Privacy Rule (§164.514 b) [27].

The survey was posted to various social media platforms such as Twitter, Facebook, and LinkedIn, as well as specific Facebook groups such as “Living With Exocrine Pancreatic Insufficiency Support Group” and in a diabetes-related group, “CGM In The Cloud Off Topic”. There were no exclusion criteria, and anyone 18 years of age or older was recruited, whether or not they had exocrine pancreatic insufficiency or any known gastrointestinal condition. The survey is still open and data collection is ongoing [28]; however, the sample for this study (n = 324 overall) was based on the first 3 weeks of data collected during November 2023. The higher participation of people with T1D is likely due to the author’s ties in the diabetes community, including online through these social networks.

### 2.2. Statistical Analysis

The original data analysis focused on comparisons between those with and without EPI, including statistical analyses and descriptive statistics to assess and understand group differences. This study added analysis of different types of diabetes sub-groups and overall. Due to non-parametric data distribution, Mann–Whitney U tests were used for comparison, alongside Cohen’s d test for effect size and Cronbach’s alpha for sub-score reliability. Predictors for different sub-scores were analyzed and ranked. Various additional statistical tests, including ANOVA, *t*-tests, and Tukey HSD, were applied to assess data distributions, variances, and group differences. Demographic statistics for each of the sub-groups with diabetes are reported to aid in characterizing the populations participating in this study to characterize differences in symptom burden with and without EPI.

## 3. Results

People with T1D had statistically significant (*p* < 0.001) differences in EPI/PEI-SS score if they have EPI (mean 100.86; SD: 48.92; min 9; max 195), mirroring the differences in people with and without EPI without T1D (*p* < 0.001). For context, the previous overall analysis observed a mean score (possible range 0–225) among people with EPI, irrespective of diabetes, of 98.11 (SD: 45.46, min 1, max 213), in contrast to a mean score of 38.86 for those without EPI (SD: 34.70, min 0, max 163). In people with type 1 and type 2 diabetes, the area under the ROC curve (AUC) was 0.86, indicating a high level of discrimination between EPI and not EPI.

There were no significant differences between people with and without diabetes who had EPI. People with T1D with EPI (n = 14) had a mean score of 100.86 (SD: 48.92). People with T1D without EPI (n = 78) had a mean score of 31.59 (SD: 28.25), which was insignificantly lower (*p* = 0.074) than that in those without T1D and without EPI (n = 91, mean 45.10, SD: 38.46).

People with T2D and EPI (n = 20) had similarly high mean total scores (103.85; SD 56.03) to those with T1D, and there was no statistical significance nor practical difference between T1D and T2D groups with EPI. Those with T2D without EPI are a small group (n = 6), and the mean score (60.33, SD 45.96) is statistically insignificantly different from the T1D without EPI group (*p* = 0.693).

**Figure 1:**
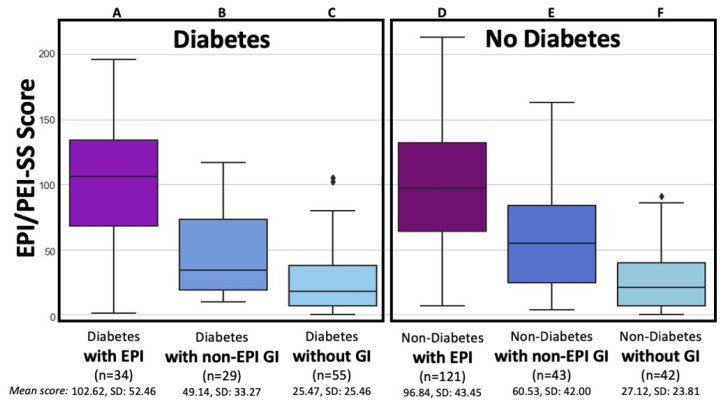
Total EPI/PEI-SS Scores by EPI, other non-EPI GI Conditions, and lack of GI conditions for People with and without Diabetes. The total EPI/PEI-SS scores is broken down among six groups categorized by the presence of diabetes and gastrointestinal (GI) condition: individuals with diabetes and EPI (Group A, n = 34, mean 102.62, SD 52.46); individuals with diabetes and other non-EPI GI conditions (Group B, n = 29, mean 49.14, SD 33.27); individuals with diabetes without any GI conditions (Group C, n = 55, mean 25.47, SD 25.46); individuals without diabetes but with EPI (Group D, n = 121, mean 96.84, SD 43.45); individuals without diabetes but with other non-EPI GI conditions (Group E, n = 43, mean 60.53, SD 42.00); and individuals without diabetes or GI conditions (Group F, n = 42, mean 27.12, SD 23.81).

**Figure 2:**
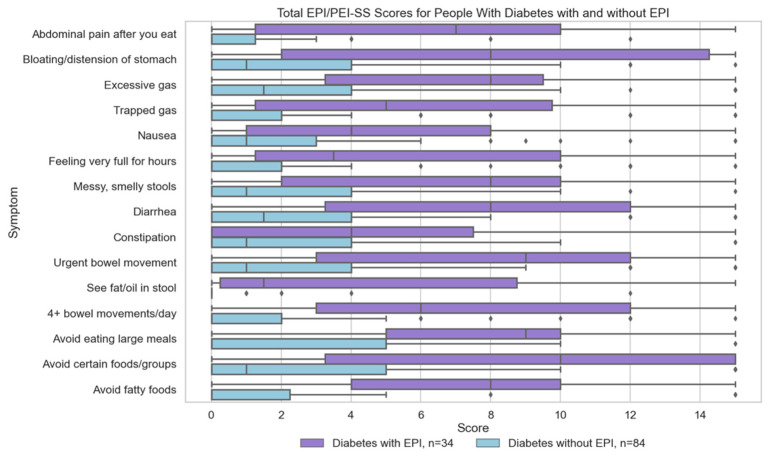
Individual Symptoms Scores for People with Diabetes With and Without EPI. Individual symptom scores for people with diabetes, distinguishing between those with Exocrine Pancreatic Insufficiency (EPI) (n = 34) and those without EPI (n = 84).

Table 1 articulates these total score descriptive statistics by diabetes sub-group; Figure 1 visualizes the score distributions in those with and without diabetes overall; and Figure 2 illustrates the spread in symptom score in people with diabetes with and without EPI.

There were statistical and practical differences among individuals with diabetes with and without non-EPI GI conditions. In the group of individuals with diabetes without EPI, there was a statistically significant difference between those with (group B) and without (group C) other GI-related conditions. This included any other GI conditions such as IBS, gastroparesis (delayed gastric emptying), celiac disease, GERD, or lactose or other food intolerances. A mean score among those with diabetes with other GI conditions (group B) was higher than those with diabetes without other GI conditions (group C) (49.14, SD: 33.27 vs. 25.47, SD 25.46; *p* < 0.001) and slightly but significantly lower (*p* < 0.001) than the group without EPI or diabetes but with other GI conditions (group E) (60.53, SD 42.00). The other GI-related conditions experienced by those with T1D and T2D, both with and without EPI, are listed in Table 2.

Individual symptom scores are over 2.5 times higher in people with diabetes with EPI than the same symptom scores in people with diabetes without EPI. Those scores are similar to those with or without EPI in people without diabetes (Table A1 and Table A2 in Appendix A). In people with diabetes, all three food-related symptoms have the highest difference when comparing mean total score of those with and without EPI (Figure 2).

**Figure 3:**
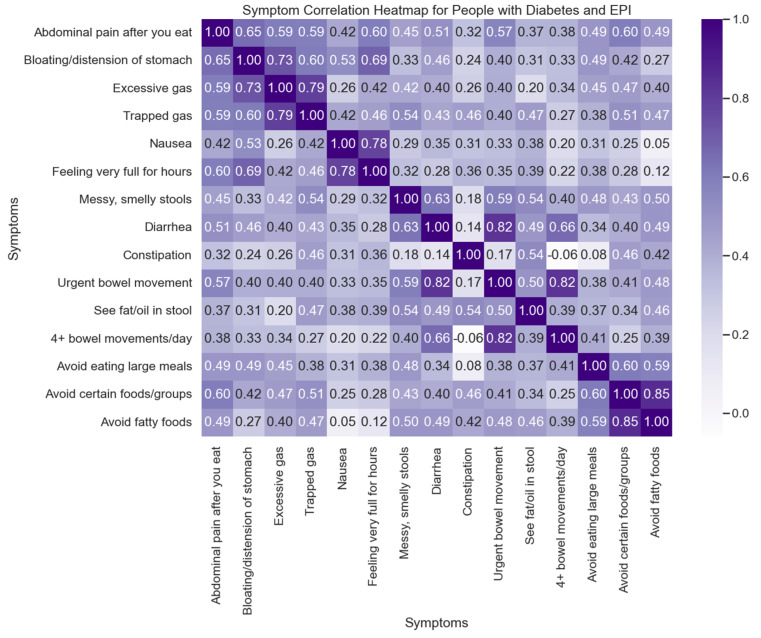
Symptom Correlation Heatmap for People with Diabetes and EPI. This heatmap illustrates the correlation between various symptoms experienced by people with diabetes who also have Exocrine Pancreatic Insufficiency (EPI). Each cell in the matrix represents the correlation coefficient between two symptoms, with the intensity of the color indicating the strength of the correlation (ranging from −1 to 1).

All people with EPI (irrespective of diabetes) exhibit a similarly high number of average symptoms (12.59), frequency score (3.06), severity score (1.79), and individual symptom score (5.48), markedly above those without EPI (regardless of diabetes, as shown in Table A3 in Appendix A). The correlation between symptoms is illustrated in Figure 3 for those with diabetes and EPI, and a lasso regression performed (Table A4 in Appendix A) further confirms the association between food-related behaviors along with bloating and feeling very full for hours as symptoms that are predictive of a high total symptom score in people with diabetes and EPI.

## 4. Discussion

The Exocrine Pancreatic Insufficiency Symptom Score (EPI/PEI-SS) may be a useful, non-invasive screening tool to aid clinicians in recognizing likely cases of EPI among people with diabetes. This sub-analysis of EPI/PEI-SS scores found that people with T1D (n = 14) and T2D (n = 20) with EPI have similar GI symptom burdens compared to those with EPI living without diabetes. People with diabetes (both Type 1 and Type 2) with EPI may have a high gastrointestinal symptom burden, even with existing use of pancreatic enzyme replacement therapy (PERT), indicating additional optimization and dose titration is needed for people with diabetes with EPI.

This gastrointestinal symptom burden is not unique to people with diabetes with EPI. PERT has previously been studied for safety and efficacy in people with diabetes [19], although optimal PERT dosing has not been studied in people with diabetes. However, numerous studies [29,30,31,32] find that fewer than 10% of studied populations are prescribed starting doses of PERT matching clinical guidelines. Real-world studies [25] have indicated a higher dose above starting guidelines is necessary to manage symptoms, matching previous clinical studies [33,34,35]. This study with the EPI/PEI-SS observed a median dose of 72,000 lipase units, yet the symptom burden was still high. This data collectively indicates that people with EPI, including people with diabetes, likely need more enzymes [6] to better match their food intake [36] (food intake was not recorded in this study). The recent AGA Clinical Guidelines on EPI affirm this, reiterating that “PERT treats food, not the pancreas” [37]. The level of elastase (e.g., whether <100 µg/g, <200 µg/g, or even above 200 µg/g, given recent studies [38]) does not determine the level of dosing required to eliminate symptoms. One promising strategy for PERT dosing is, instead of fixed dosing, using a ratio similar to the insulin-to-carb ratio, a concept which may be familiar and comfortable to people living with diabetes who have EPI [39]. This can usually be done with a single ratio of lipase to fat consumed, although people with diabetes may also be sensitive to protein as well and benefit from monitoring their protease-to-protein ratio if they are having trouble with achieving symptom resolution solely with focusing on lipase-based dosing.

The influence of a high GI symptom burden (indicating likely EPI) on glycemic outcomes among people with diabetes should be further studied. A previous n = 1 with T1D case study [40] evaluated glycemic outcomes before and after the onset of pancreatic enzyme replacement therapy (PERT), showing that untreated EPI likely contributes to above-range glycemic excursions post-prandially, even with automated insulin delivery (AID) and above-goal overall TIR and ideal HbA1c. As-of-yet unpublished case reports from endocrinologists suggest that otherwise unexplainable glycemic variability and non-optimal glucose outcomes may be resolved by starting PERT in patients with low elastase levels, even in those who do not report overt gastrointestinal symptoms. Endocrinologists and other care providers who test elastase levels among PWD based on glycemic variability, rather than initial presentation of high gastrointestinal symptom burden, may be able to nonetheless identify a still above-average GI symptom burden among these patients, although they may not present with a high GI symptom burden as a primary complaint. To date, other symptom surveys for EPI in people with diabetes have not been widely tested, and symptom scores such as the PEI-Q [20,21,22] or EPI-SQ [21], designed for and validated primarily for populations with chronic pancreatitis and cystic fibrosis, have failed to reproduce in populations with diabetes [23], which may play a role in the clinical perception of a lower GI symptom burden. On the other hand, the lack of overt awareness of GI-related symptom burden at early stages has been hypothesized [14] to be a result of PWD having already adjusted their behaviors, including dietary choices, in response to glucose levels, thus intuitively reducing the longer-term GI symptom burden. These subtle behavioral and dietary choice adjustments may happen over the course of years among people with diabetes, given that recent studies show adequate elastase levels at the time of type 1 diabetes diagnosis [41]. This is further supported in the current study, which found higher scores for food-related behaviors among people with diabetes with EPI compared to those without EPI (with or without diabetes). This is also supported by a study, not specific to people with diabetes, that observes that many individuals with fecal elastase levels 200–500—traditionally considered to be not representative of EPI—benefit from PERT and concludes that there may be a graded, gradual decline in elastase that correlates with the onset of GI symptoms and strict, distinct cutoffs may no longer be appropriate for diagnosing EPI [38].

People with diabetes who present with gastrointestinal symptoms should be screened for exocrine pancreatic insufficiency given the high prevalence of EPI, which is likely above and beyond that of celiac and gastroparesis among PWD [2]. Diabetes care providers should initiate elastase screening as needed for patients who mention GI symptoms [42], including symptoms other than diarrhea and steatorrhea. This overall study with the EPI/PEI-SS and sub-analysis among people with diabetes showed symptoms ranging from constipation (albeit less frequent) to food avoidance-related symptoms and painful bloating, gas, and abdominal pain. If desired, diabetes care providers could administer the EPI/PEI-SS [43] prior to elastase screening as well.

Diabetes care providers can and should review PERT dosing regularly with people with diabetes and already-diagnosed EPI, and assess GI symptoms of people with diabetes, even for those with longer duration EPI. The data from this sub-analysis shows a too-high symptom burden of EPI, despite taking PERT (a third more symptoms and averaging more than once per week, double the frequency as well as double the severity for any individual symptom compared to those without EPI), for people living with diabetes. Duration of EPI did not associate with a lower symptom score, as one might predict, indicating ongoing need for support with dose titration. The EPI/PEI-SS should be further studied to assess whether it is a useful aid for tracking individual symptom changes over time for people with diabetes and EPI, especially for those with subclinical or mild to moderate EPI represented by borderline or 100–200 µg/g fecal elastase, which may be harder to correlate with other pancreatic function tests in people with diabetes [44], not only for type 1 but also for type 2 diabetes [45].

Strengths of this study include a real-world dataset of gastrointestinal symptom data among people with and without diabetes with EPI and other gastrointestinal conditions, and use of a novel metric that evaluates common symptoms by frequency and severity. This may aid clinicians in more effectively screening people with diabetes with gastrointestinal symptoms for EPI. This study’s limitations include the fact that it uses real-world data from people with diabetes who found and participated in this online survey through social media/social networks, so the participant pool may not be representative of the general population of people with diabetes. It also was not powered for a pre-specified effect. As such, the EPI/PEI-SS should be further studied in offline populations of people living with diabetes identified in clinical settings to determine whether these results are representative and reflective of people with diabetes. This should include consideration of other imaging as well as an assessment of the timeline of diagnoses for EPI, diabetes, and any other overlapping gastrointestinal conditions. Attention should also be paid to additional diabetes-related or other medications with known gastrointestinal side effect profiles, which was not collected in the current study. The lower scores of people with diabetes with other GI conditions compared to those without diabetes and other GI conditions could be an artifact of very motivated people with diabetes (e.g., present and active also in social media related to diabetes); or it could be an artifact of the fact that people with diabetes may be more aware of their body and intuitively adjusting dietary choices and behaviors in order to avoid GI symptoms and/or based on feedback related to glucose levels. Yet those with EPI with and without diabetes did not have statistically significant nor practical differences in mean scores, so this may not heavily influence the findings of the EPI/PEI-SS among people with diabetes with EPI or suspected EPI. This can be confirmed with subsequent studies designed to adjust for these possible biases, including studies designed for assessing the ability of the EPI/PEI-SS to support individuals’ evaluation of symptom changes over time with PERT titration. There may also be some cases of undiagnosed EPI or other gastrointestinal conditions in the group who identified as not having EPI, given the number of outlier data points on total score and individual symptom burden in the non-EPI group.

## 5. Conclusions

Exocrine pancreatic insufficiency is prevalent yet remains under-diagnosed among people with diabetes, and even after diagnosis with EPI, the symptom burden among people with diabetes living with EPI (PEI) remains high. This is primarily considered to be gastrointestinal symptom burden, but emerging data suggests glycemic variability and outcomes may also be influenced by non-optimal digestion, with or without overt gastrointestinal symptoms. The EPI/PEI-SS showed promise with initial sub-analysis among people with and without diabetes to differentiate the symptom burden of EPI, distinct from other GI conditions and non-condition-based everyday GI symptoms. People with diabetes and EPI have a similar symptom burden compared to people without diabetes who have EPI. Diabetes care providers can play a role in evaluating PERT dosing and efficacy and improving symptom burden for people with diabetes and EPI. Clinicians who treat people with diabetes should also help screen people with diabetes with GI symptoms for EPI and be aware that some people with glycemic variability challenges may also be candidates for EPI screening. The EPI/PEI-SS [43] may be one tool that can aid providers in evaluating people with gastrointestinal symptoms for additional EPI screening such as fecal elastase testing. Further studies are needed to correlate EPI/PEI-SS result levels with elastase at a population level and to determine whether the EPI/PEI-SS can also support people with diabetes for individualized symptom tracking over time with pancreatic enzyme replacement therapy titration.

## Figures and Tables

**Table 1:** Total Scores for Those with T1D and T2D With and Without EPI.

Group	Count	Mean	Median	Minimum	Maximum	Standard Deviation (SD)
EPI with T1D	14	100.86	108.00	9.00	195.00	48.92
Non-EPI with T1D	78	31.59	21.50	0.00	110.00	28.25
EPI with T2D	20	103.85	104.50	1.00	196.00	56.03
Non-EPI with T2D	6	60.33	72.50	0.00	117.00	45.96

**Table 2:** Count of Individuals with Other Gastrointestinal-Related Conditions.

Condition	T1D EPI	T1D Non-EPI	(T1D Total)	T2D EPI	T2D Non-EPI	(T2D Total)
IBS	4	6	(10)	9	1	(10)
Lactose intolerance	3	5	(8)	5	2	(7)
GERD	1	6	(7)	7	0	(7)
Other food intolerances	2	4	(6)	3	0	(3)
Gastroparesis	4	6	(10)	1	0	(1)
Celiac disease	2	3	(5)	1	1	(2)

## Data Availability

Raw data (anonymized) is available upon request to the corresponding author.

## Abbreviations

The following abbreviations are used in this manuscript:

T1D	Type 1 diabetes
T2D	Type 2 diabetes
GI	Gastrointestinal
EPI	Exocrine Pancreatic Insufficiency
PEI	Pancreatic Exocrine Insufficiency
PERT	Pancreatic Enzyme Replacement Therapy

## Appendix A. A Note on Non-EPI GI Conditions and Symptom Burden Among People with Diabetes

Interestingly, within the non-EPI cohort, individuals with T1D exhibited statistically significant lower EPI/PEI-SS scores compared to those without diabetes, indicating an overall lower GI symptom burden on average. This may be mediated by individual behavior which may be intuitively lowering the GI symptom burden, or it may be a result of the sample selection for this survey. This pattern was also evident when focusing specifically on individuals with GI-related conditions but without EPI: those with T1D and other GI conditions had significantly lower mean EPI/PEI-SS scores (43.65) compared to those without T1D but with other GI conditions (90.16, *p* < 0.001).

**Table A1:** Mean Individual Symptom Scores for Groups with and without Diabetes and With and Without EPI.

	Diabetes with EPI				
Symptom	Mean	Median	SD	Min	Max
Abdominal pain after you eat	6.41	7	5.11	0	15
Bloating/distension of your stomach	7.82	8	5.63	0	15
Excessive gas	7.12	8	4.89	0	15
Trapped gas	6	5	5.32	0	15
Nausea	5.44	4	5.12	0	15
Feeling very full after you eat	6.09	3.5	5.53	0	15
Messy, smelly stools	6.97	8	4.99	0	15
Diarrhea	7.91	8	5.08	0	15
Constipation	4.62	4	4.81	0	15
Urgent bowel movement	8.29	9	5.34	0	15
See fat or oil in stool	4.32	1.5	4.64	0	15
4 or more bowel movements per day	7.38	6	5.14	0	15
Avoid eating large meals	8.09	9	4.8	0	15
Avoid certain foods or food groups	8.94	10	5.73	0	15
Avoid fatty foods	7.21	8	4.95	0	15
	**Diabetes without EPI**				
**Symptom**	**Mean**	**Median**	**SD**	**Min**	**Max**
Abdominal pain after you eat	1.02	0	2	0	12
Bloating/distension of your stomach	2.62	1	3.74	0	15
Excessive gas	3.12	1.5	4.31	0	15
Trapped gas	2.17	0	3.74	0	15
Nausea	2.26	1	3.37	0	15
Feeling very full after you eat	2.08	0	3.71	0	15
Messy, smelly stools	2.33	1	3.22	0	15
Diarrhea	2.8	1.5	3.81	0	15
Constipation	2.39	1	3.36	0	15
Urgent bowel movement	3.07	1	4.22	0	15
See fat or oil in stool	0.36	0	1.48	0	12
4 or more bowel movements per day	2.02	0	3.44	0	15
Avoid eating large meals	2.29	0	3.31	0	15
Avoid certain foods or food groups	3.49	1	4.84	0	15
Avoid fatty foods	1.62	0	3.25	0	15
	**No Diabetes with EPI**				
**Symptom**	**Mean**	**Median**	**SD**	**Min**	**Max**
Abdominal pain after you eat	5.67	4	5.23	0	15
Bloating/distension of your stomach	7.14	6	5.29	0	15
Excessive gas	7.31	8	5	0	15
Trapped gas	5.23	4	4.96	0	15
Nausea	4.46	2	4.84	0	15
Feeling very full after you eat	5.8	4	5.19	0	15
Messy, smelly stools	7	6	4.75	0	15
Diarrhea	7.87	8	5.41	0	15
Constipation	3.55	2	4.26	0	15
Urgent bowel movement	7.62	8	5.16	0	15
See fat or oil in stool	4.79	4	4.7	0	15
4 or more bowel movements per day	5.79	4	5.35	0	15
Avoid eating large meals	7.71	8	4.84	0	15
Avoid certain foods or food groups	9.07	10	5.39	0	15
Avoid fatty foods	7.84	8	5.32	0	15
	**No Diabetes without EPI**				
**Symptom**	**Mean**	**Median**	**SD**	**Min**	**Max**
Abdominal pain after you eat	2.49	1	3.9	0	15
Bloating/distension of your stomach	3.73	2	4.2	0	15
Excessive gas	3.88	2	3.87	0	15
Trapped gas	2.74	1	3.49	0	15
Nausea	2.74	0	4.18	0	15
Feeling very full after you eat	2.13	0	3.58	0	15
Messy, smelly stools	2.59	1	3.49	0	15
Diarrhea	3.72	2	3.91	0	15
Constipation	3.54	2	4.22	0	15
Urgent bowel movement	3.75	2	4.26	0	15
See fat or oil in stool	0.94	0	2.45	0	15
4 or more bowel movements per day	2.04	0	3.44	0	15
Avoid eating large meals	2.82	1	3.57	0	15
Avoid certain foods or food groups	4.36	3	5.07	0	15
Avoid fatty foods	2.54	1	3.63	0	15

**Table A2:** Three Top and Bottom Mean Individual Symptom Scores for Groups with and without Diabetes and With and Without EPI.

	Diabetes with EPI		Diabetes Without EPI		
	Symptom	Mean		Symptom	Mean
TOP 3	Avoid certain food/groups	8.94	TOP 3	Avoid certain food/groups	3.49
	Urgent bowel movement	8.29		Excessive gas	3.12
	Avoid eating large meals	8.09		Urgent bowel movement	3.07
BOTTOM 3	Nausea	5.44	BOTTOM 3	Avoid fatty foods	1.62
	Constipation	4.62		Abdominal pain after you eat	1.02
	See fat or oil in stool or on toilet paper	4.32		See fat or oil in stool or on toilet paper	0.36
	**No Diabetes with EPI**		**No Diabetes without EPI**		
	**Symptom**	**Mean**		**Symptom**	**Mean**
TOP 3	Avoid certain food/groups	9.07	TOP 3	Avoid certain food/groups	4.36
	Diarrhea	7.87		Excessive gas	3.88
	Avoid fatty foods	7.84		Urgent bowel movement	3.75
BOTTOM 3	See fat or oil in stool or on toilet paper	4.79	BOTTOM 3	Feeling very full for hours after you eat	2.13
	Nausea	4.46		4 or more bowel movements per day	2.04
	Constipation	3.55		See fat or oil in stool or on toilet paper	0.94

**Table A3:** Average Number of Symptoms in Those With and Without Diabetes And EPI.

	Group	Average Number of Symptoms	Average Frequency Score	Average Severity Score	Average Individual Symptom Score
(EPI)	Diabetes with EPI	12.59	3.06	1.79	5.48
	No Diabetes with EPI	12.33	3	1.71	5.13
(Non-EPI)	Diabetes without EPI	7.36	1.4	0.8	1.12
	No Diabetes without EPI	8.94	1.7	1.03	1.75

**Table A4:** Predictors of Higher EPI/PEI-SS Score in Those With and Without Diabetes And EPI.

	EPI		No EPI	
	Diabetes with EPI	No Diabetes with EPI	Diabetes without EPI	No Diabetes without EPI
Avoid certain foods/groups	5.67	5.37	4.81	5.04
Bloating/distension of stomach	5.51	5.28	3.77	4.21
Feeling very full for hours	5.49	5.17	3.69	3.55
Trapped gas	5.32	4.95	3.7	3.46
Urgent bowel movement	5.31	5.13	4.17	4.24
Abdominal pain after eating	5.05	5.2	1.99	3.87
Diarrhea	5	5.37	3.8	3.87
4+ bowel movements/day	5	5.32	3.42	3.39
Nausea	4.97	4.82	3.34	4.14
Messy, smelly stools	4.88	4.71	3.18	3.45
Avoid fatty foods	4.85	5.29	3.2	3.6
Excessive gas	4.75	4.95	4.27	3.85
Avoid eating large meals	4.69	4.82	3.27	3.55
Constipation	4.66	4.21	3.32	4.17
See fat/oil in stool	4.6	4.67	1.45	2.43
R^2^ Score	0.999990	0.999997	0.999985	0.999996
